# Vesicoureteral reflux: surgical and endoscopic treatment

**DOI:** 10.1007/s00467-006-0415-9

**Published:** 2007-09-01

**Authors:** Nicola Capozza, Paolo Caione

**Affiliations:** Department of Pediatric Urology, “Bambino Gesù” Children’s Hospital, Piazza S. Onofrio, 4-00165 Rome, Italy

**Keywords:** Vesicoureteral reflux, Endoscopy, Surgery, Laparoscopy, Uropathy

## Abstract

The optimal management of vesicoureteral reflux (VUR) is quite controversial. For many years, only antibiotic prophylaxis and open surgery were considered possible options. Since the first descriptions in the early 1980s, endoscopic treatment (ET) has gained popularity and is now considered a valid alternative both to open surgery and antibiotic prophylaxis. Many surgical antireflux techniques have been described in the past 50 years. The general principle of reflux surgery, usually defined as ureteric reimplantation, is elongation of the submucosal ureteral tunnel with creation of a flap-valve mechanism. The antireflux operation can also be carried out laparoscopically, either extravesically or intravesically (pneumovesicum). Open surgery is associated with a high success rate (>95%) regardless of the technique adopted. However, because it is invasive, it is limited to selected cases. Laparoscopic technique is less invasive, but the mean operative time is much longer and results depend significantly on the learning curve. ET involves injecting material endoscopically into the submucosal space under the ureteric orifice. It is associated with a good success rate (about 80% after one injection). Advantages of this minimally invasive treatment include repeatability and the fact that postoperative complications are rare. With a second injection, after few months if needed, the success rate of ET approaches that of open surgery. Our 20-year experience in ET is described in detail in this paper, as this technique has changed the management algorithm for VUR dramatically.

## Introduction

Vesicoureteral reflux (VUR) is the most common uropathy in children (0.4–1.8% of pediatric population); nevertheless, its optimal management remains controversial. Until the 1980s, treatment guidelines recommended antibiotic prophylaxis (AP) as “therapy” for mild-grade reflux (I–II). AP was also indicated as initial therapy for grades III–IV. Open surgery was recommended for patient with high-grade (IV–V) or persistent (any grade) reflux [1].

Endoscopic treatment (ET) of reflux by means of subureteral injection of bulking materials was first described by Matouschek in 1981 and further developed by Puri and O’Donnell [2–4]. Since then, ET has gained popularity and has proved successful in a high percentage of cases. ET is now considered a valid alternative to open surgery and AP [5]. As a result of the improvement in ET, open surgery is indicated only in very selected cases.

This paper presents an overview of the main surgical options and the endoscopic approach, with particular emphasis on ET, as this technique has changed the management algorithm for VUR dramatically. Our 20-year experience in this field is also described.

### The refluxing ureter

According to the Stephens’ theory, the primary anomaly that results in VUR is an ectopic ureteral bud during morphogenesis [6]. As a consequence, the ureteral orifice is in a lateral position, with an intravesical ureter shorter than normal. Also, the configuration and competency of the ureteral orifice are altered [7]. The aim of both surgery and ET for VUR is elongation of the submucosal ureteral tunnel with creation of a flap-valve mechanism.

### Open surgery

**Fig. 1:**
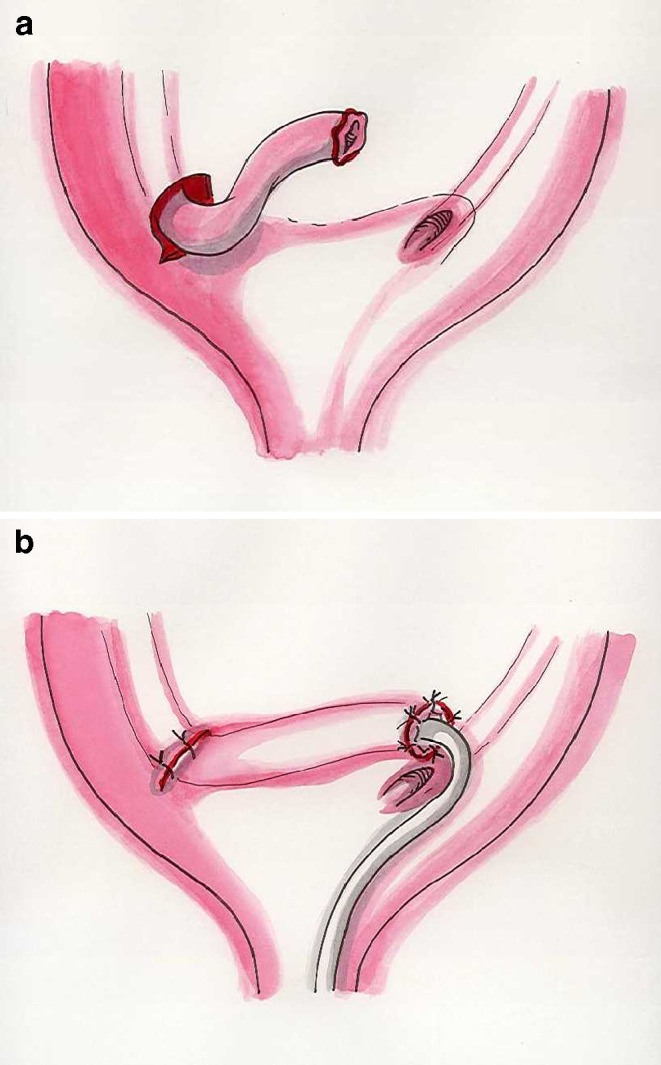
**a** Cohen procedure. The ureter is mobilized intravesically. **b** Ureteral orifice at the end of the submucosal tunnel

- The Hutch technique (1952) first introduced the concept of creating an antireflux valve by elongation of the intravesical portion of the ureter. The detrusor is incised laterally to the original hiatus and then closed under the ureter. This technique does not allow correction of any kinking or any tapering of the ureter [8].
- One of the most widely used surgical techniques was described by Politano and Laedbetter in 1958. The ureter is freed from the trigone and mobilized intravesically. It is then passed extravesically and brought inside the bladder through a new hiatus, which positioned superior and lateral to the original orifice [9].
- The Lich–Gregoir (1961–1964) is an extravesical technique. The juxtavesical ureter is isolated, and the detrusor is incised superior and lateral to the ureteral hiatus, creating a submucosal bed of the ureter; the detrusor is then sutured over the ureter [10, 11].
  This technique has some advantages: it does not require opening the bladder, it is quick, and there is no need for stenting. It is widely used in renal transplant.
- The Glenn–Anderson advancement technique (1967) is most applicable when there is enough space to advance the intravesical ureter. The submucosal tunnel is created from the ureteral hiatus distally and medially toward the bladder neck [12].
  A modification of the Glenn–Anderson technique (1978) is a more extensive dissection of the ureter, with a resulting larger hiatus. Subsequent closure of the distal part of the hiatus allows creation of a longer tunnel for the ureter [13].
- The Cohen procedure (1975) is the most widely used technique in the world. The ureters (the procedure is often bilateral) are mobilized intravesically, and two separate submucosal tunnels are created so that each ureter opens on the opposite side from its hiatus [14] (Fig. 1a,b).
  The only disadvantage of the Cohen procedure is in the event a ureteral retrograde catheterization becomes necessary (for instance, to remove stones) due to the orientation of the ureteral orifices.

### Laparoscopic antireflux operations

Laparoscopic reimplantation of the ureters can be carried out either extravesically or intravesically. Extravesical reimplantation does not differ from the “open” Lich–Gregoir method. The mean operative time is much longer: in a recent series, it took 1.75 h for unilateral and 3.75 h for bilateral VUR versus 0.50 and 1.00 h for open the Lich–Gregoir technique [15].

Endoscopic intravesical ureteral mobilization and Cohen’s cross-trigonal reimplantation under carbon dioxide insufflation of the bladder (pneumovesicum) was proposed by Yeung in 2005. The mean operating time in his series was 136 min [16]. This technique is certainly less invasive than open surgery but much more invasive than endoscopic injection therapy.

### Endoscopic injection therapy

#### Injection technique

**Fig. 2:**
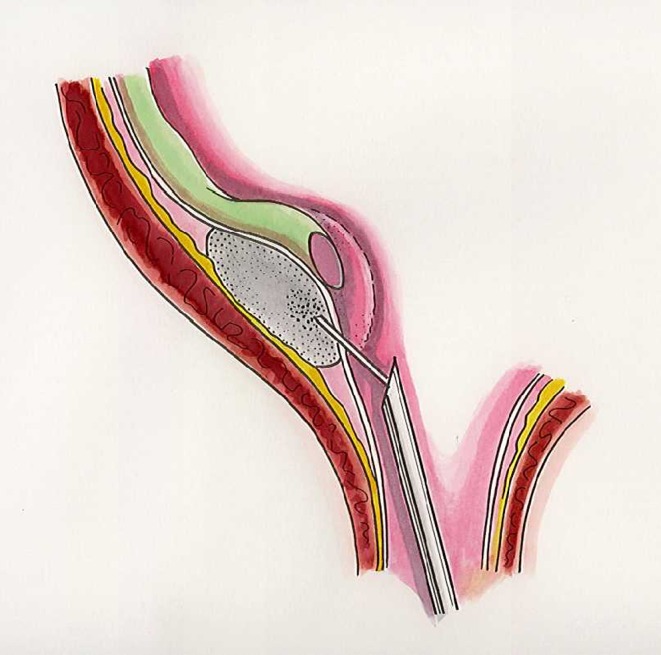
Endoscopic subureteral injection

The ET technique was originally described by Puri and O’Donnell for subureteral polytetrafluoroethylene injection and is referred to as the STING operation [4]. The most common cystourethroscopes are the Wolf–O’Donnell 10 Ch (Richard Wolf GMBH, Khittlingen Germany), the Storz 10 Ch (Storz, Tuttlingen, Germany), and the Wolf 14 Ch. The optic lens varies from 5° to 30°. We recently began using a new cystoscope, the Wolf 8/9,8 Ch, which is particularly effective in young infants as it has a very thin distal section (8 Ch). The material is injected through a 23-gauge endoscopic needle. According to the original technique, the needle is inserted a few millimeters below the ureteral orifice, and the material is injected into the terminal submucosal tract of the ureter (Fig. 2). At the end of the procedure, a volcano-like projection with the ureteral orifice on top should be visible.

Technical adjustments are necessary in some instances, particularly in cases of ET for VUR after failed surgery [17]. In 2005, Kirsch and Scherz presented a modification of the technique as an evolution of the STING procedure, named the hydrodistention implantation technique (HIT). This technique is based on two concepts: hydrodistention of the ureteral orifice and submucosal intraureteral implantation of the material. With this technique, the needle is placed within the ureteral tunnel, and the injection is performed into the submucosal intraureteral space along the entire length of the detrusor tunnel [18]. In our experience, this technique has proved useful in high-grade reflux with a short tunnel when an intraureteral injection is feasible even without hydrodistention. In low-grade VUR, we give preference to the standard technique, which avoids hydrodistention and the consequent risk of seeding the kidney with bacteria. The amount of injectable material varies from 0.1 to 1.5 ml, depending on the experience of the operators. With greater experience, less material can be used to achieve a satisfactory implant configuration.

### Twenty-year experience with endoscopic treatment

#### (a) Patients and methods

From January 1986 to June 2005, 1,732 patients (2,455 refluxing ureteral units) ranging from 5 months to 22 years (average 28 months) in age underwent ET for grades II–V VUR. The majority of patients (1,608 patients; 2,293 ureters) had primary reflux. Our series included reflux associated with a duplex system (58 patients; 62 ureters), neurogenic bladder (20 patients; 37 ureters), posterior urethral valves (18 patients; 24 ureters), and secondary to failed surgery (28 patients; 39 ureters). Because voiding dysfunction represents a possible negative factor in the success of ET, all children with VUR older than 3 years of age were evaluated using a micturition questionnaire, uroflowmetry, and measurement of postvoid residual urine. Patients with voiding dysfunction were treated at least 6 months before ET with anticholinergics and/or micturition rehabilitation.

Polytetrafluoroethylene was used in the initial 14 cases. After 1989, glutaraldehyde cross-linked bovine collagen was used in 442 cases, and since 1995, dextranomer/hyaluronic acid (Dx/HA) was used in 1,276 cases (1,811 ureters).

Children were discharged 24 h after treatment. AP was continued for 1 month postoperatively.

Follow-up consisted of periodic urinalysis, renal and bladder ultrasound 1 month after treatment, and, in our initial experience, micturition cystourethrogram (MCUG) 3 and 12 months after treatment. In the last 7 years, we performed a single MCUG 3–6 months after treatment. In children having acquired urinary control and without urinary tract infection (UTI), mercaptoacetyltriglycine-3 (MAG3) renal scan with indirect voiding cystogram has recently been preferred to traditional fluoroscopic MCUG. Persistence of VUR and the first posttreatment MCUG were considered early failure of ET. Recurrence of VUR after a successful ET was considered late failure. In both situations, a second treatment was carried out.

#### (b) Results

After one injection, MCUG showed no VUR or grade I in 79% of ureters. The success rate was 91%, 78%, and 62% for grades II, III, and IV–V, respectively.

After a second injection, the success rate increased to 91%. The success rate did not show significant difference in primary and secondary reflux. A significant improvement in the success rate was noted in the most recent years compared with the previous years. A transient ureteric obstruction was observed in eight cases (0.4%). The only major complication was a prolonged hematuria requiring blood transfusion in one patient.

In cases with early failure (21%), the implanted material was not evident at the second ET (30% of cases); this finding could be related with an incorrect injection technique. Late failure occurred in 3% of the series, and the most common finding was implant displacement (45% of cases with late failure), possibly due to a concomitant bladder dysfunction.

## Discussion

In recent years, there have been major advances in ET, mainly in relation to new injectable materials and improved endoscopic instruments and technique [5]. For about 15 years, ET has been performed using mainly polytetrafluoroethylene, silicone, and bovine collagen, but concerns about their safety and efficacy have precluded their widespread use. Dx/HA copolymer has proved to be safe and much more effective than AP [19]. To date, no contraindications are considered for ET for VUR.

Some trials compared different materials for subureteric injection to correct VUR (polydimethylsiloxane versus Dx/HA; different concentrations of cross-linked collagen). There was no significant difference in the results at long-term follow-up [20, 21].

Availability of the right endoscopic instruments is of primary importance to achieve good results and allow application of the technique even to infants (>6 months).

During the last 5 years, results of ET for VUR have constantly improved due to the above-described technical adjustments. Learning curve as been postulated as an important factor in the success rate, but further studies are necessary to clarify the role of the surgeon as a determinant of injection success. The most recent series showed an overall success rate of about 90%, and improvement was more evident in VUR grade IV [18]. Since this success rate approaches that of open surgery, there may be a rationale for eliminating the standard postoperative micturition cystourethrogram.

Although VUR is a common disorder in the pediatric population, there is controversy about reflux management. As a result of the advances in ET, the 1997 American Urological Association (AUA) guidelines are being reevaluated to include this procedure in the management of VUR [22, 23]. To date, the three main options for VUR are open surgery, AP, and ET with injectable materials. Few randomized comparative studies have been performed in recent years to assess the optimal management of VUR. If the goal is to prevent renal damage, there is no evidence of better results with one treatment than another. However, if the goal is to cure VUR and avoid daily antibiotics and yearly cystographies, there is no doubt that both surgery and ET are much more effective than AP [19, 23].

Some studies have been performed to assess the cost and outcome of ET for VUR compared with antibiotics and surgery [24]. Conclusions of these studies are that ET for VUR appears to be cost effective when compared with open surgery; cost-effectiveness is less obvious compared with AP (or simple observation). Human costs are more difficult to measure. If we consider invasiveness and disadvantages of the different options, ET needs about 10 min of general anesthesia, is a 1-day procedure, and possible complications are usually limited to a mild dysuria and temporary ureteric obstruction, which do not require therapy. Open surgery needs 60–90 min of general anesthesia, entails an abdominal incision, requires about 5 days in hospital and 3 weeks for full recovery, and causes postoperative pain and possible major complications: i. e., ureteric obstruction, bleeding, contralateral reflux, and bladder dysfunction, mainly when the operation is performed in the first year of life. In our opinion, open surgery is indicated in very complex situations, such as reflux associated with large Hutch diverticulum or symptomatic patients with VUR persistent after one to two endoscopic sessions.

## Conclusions

In our opinion, the advent of ET has changed the algorithm of reflux management in children. ET is minimally invasive, can be performed as 1-day surgery (or even as an outpatient procedure), and has very low morbidity. Currently used injectable materials are safe and ensure long-term permanence at the site of injection. The success rate of ET is high if compared with long-term prophylaxis and due to continuous improvements of materials, instruments, and technique, it is approaching that of open surgery. ET results are satisfactory even in complex anatomical situations. On the basis of the above considerations, we propose ET as the first-line option for most cases of VUR. ET can represent a useful alternative to AP in low-grade reflux and to open surgery in high-grade reflux. Long-term prophylaxis, intermittent antibiotic therapy, or open surgery can be reserved for selected cases, mainly after failure of ET.

**QUESTIONS** (answers appear following the references)

1. What is the general principle of the VUR surgery?
  A. Narrowing of the ureteral orifice
  B. Elongation of the submucosal tunnel
  C. Mobilization of the distal ureter
2. What is the possible disadvantage of the Cohen procedure?
  A. Ureteral retrograde catheterization may be difficult
  B. Stone formation
  C. Difficult to be performed
3. Is endoscopic treatment contraindicated in neurogenic bladder?
  A. Yes
  B. No
  C. Only in case of intermittent catheterization
4. Laparoscopic antireflux operations can be carried out:
  A. Only extravesically
  B. Only intravesically
  C. Either extra- or intravesically
5. The overall success rate of endoscopic treatment for VUR is about:
  A. 80%
  B. 60%
  C. 40%

Answers:

1. B
2. A
3. B
4. C
5. A
